# Innovative Solutions for State Medicaid Programs to Leverage Their Data, Build Their Analytic Capacity, and Create Evidence-Based Policy

**DOI:** 10.5334/egems.311

**Published:** 2019-08-05

**Authors:** Lauren Adams, Susan Kennedy, Lindsay Allen, Andrew Barnes, Tom Bias, Dushka Crane, Paul Lanier, Rachel Mauk, Shamis Mohamoud, Nathan Pauly, Jeffrey Talbert, Cynthia Woodcock, Kara Zivin, Julie Donohue

**Affiliations:** 1 AcademyHealth, US; 2 West Virginia University School of Public Health, US; 3 Virginia Commonwealth University, US; 4 The Ohio State University, US; 5 University of North Carolina Chapel Hill, US; 6 The Hilltop Institute, US; 7 University of Kentucky College of Pharmacy, US; 8 University of Michigan Medical School, US; 9 University of Pittsburgh Graduate School of Public Health, US

**Keywords:** Medicaid, Health Information Exchange, Research Networks, Quality Improvement

## Abstract

As states have embraced additional flexibility to change coverage of and payment for Medicaid services, they have also faced heightened expectations for delivering high-value care. Efforts to meet these new expectations have increased the need for rigorous, evidence-based policy, but states may face challenges finding the resources, capacity, and expertise to meet this need. By describing state-university partnerships in more than 20 states, this commentary describes innovative solutions for states that want to leverage their own data, build their analytic capacity, and create evidence-based policy. From an integrated web-based system to improve long-term care to evaluating the impact of permanent supportive housing placements on Medicaid utilization and spending, these state partnerships provide significant support to their state Medicaid programs. In 2017, these partnerships came together to create a distributed research network that supports multi-state analyses. The Medicaid Outcomes Distributed Research Network (MODRN) uses a common data model to examine Medicaid data across states, thereby increasing the analytic rigor of policy evaluations in Medicaid, and contributing to the development of a fully functioning Medicaid innovation laboratory.

## Introduction

Historically, states have leveraged flexibility in the administration of Medicaid benefits to address the needs of their populations and policy priorities. Through the implementation of Medicaid waivers and demonstrations, which allow states to deviate from federal law and test alternative approaches to pay for and deliver care, Medicaid agencies have accelerated their role as the country’s laboratories for questions around how to sustainably improve insurance coverage, increase access and enhance care quality [1,2]. As states have embraced this additional flexibility to change coverage of and payment for services, they have also faced heightened expectations from their own state leadership as well as the Centers for Medicare and Medicaid Services (CMS) for delivering high-value care, which has increased the need for rigorous, evidence-based policy and program analyses.

Researchers have long struggled with the ability to make scientific findings accessible and useful to policymakers in a timely, effective, and efficient manner that allows for practical application [3]. A 2014 AcademyHealth survey asked state government officials about barriers to using evidence to inform policy. Findings indicated that state agency staff do not use evidence because it is not timely (48 percent) and not relevant (41 percent) [4]. The survey responses also highlighted the importance of state-level data collection, and the need for enhanced analytic capability and expertise. In another AcademyHealth survey of Medicaid agency staff, respondents pointed out how the lack of uniform data collection and reporting practices makes comparative studies across states overly complex and resource-intensive [5].

AcademyHealth, a non-profit professional association for health services and policy researchers, supports the production and use of evidence to inform policy and practice to improve health and health care. As the home for both the Medicaid Medical Director Network (MMDN) and State-University Partnership Learning Network (SUPLN), AcademyHealth has been directly engaged with state Medicaid clinical leaders and academic researchers on policy development and evaluation. This commentary describes innovative solutions for states that want to leverage their own data, build their analytic capacity, and create evidence-based policy.

## The State-University Partnership Learning Network (SUPLN)

State Medicaid agencies, often facing budget constraints that limit their ability to invest in analytic infrastructure, may collaborate with state university researchers to provide analytic support related to Medicaid programs.

Established in 2014, the SUPLN supports partnerships between state governmental entities, such as Medicaid agencies, and their state university research partners. The SUPLN aims to promote the use of evidence in state and agency policy and programmatic decision making, and focuses on transforming delivery of care to Medicaid enrollees. The SUPLN consists of 27 state-university partnerships across 23 states, with a few state agencies partnering with multiple universities in their states. With support from the Patient Centered Outcomes Research Institute (PCORI), AcademyHealth manages the SUPLN, facilitating dialogue and peer-to-peer learning to spread best practices. SUPLN is unique from other learning networks given its members’ role in state Medicaid policy making. More recently, AcademyHealth has focused on coordinating and promoting cross-state analyses of Medicaid data. Each member’s historical knowledge for their own state’s Medicaid program is powerful alone, but the trusted relationships between members have resulted in expanded insights into similarities and differences across Medicaid programs and policies.

Individual state-university partnerships offer unique value because of the central role they serve in conducting Medicaid policy analysis. Through formal contractual relationships, university partners work routinely with their state’s Medicaid administrative data (i.e., Fee-for-Service [FFS] Claims data, Managed Care Organization encounter data, and, in some cases, Children’s Health Insurance Program [CHIP] datasets), some hosting Medicaid data on-site at their universities and others having direct access through the Medicaid agency’s portal. The benefit of this direct, on-going access is the availability of timely Medicaid data with potential for linkages to other state data sources (e.g., vital stats, housing). This partnership structure is mutually advantageous. State agencies have access to researchers with a broad range of specialized policy, health services research, and methodologic expertise that is not typically available through state employees. This expertise is also obtained in a cost-efficient manner because states, through their contracts with university partners, are eligible for federal financial match for qualified administrative expenditures related to research and evaluation. Through these partnerships, researchers obtain access to a rich source of data for studying vulnerable populations, funding to support policy-relevant research, and the opportunity to collaborate with state policymakers to conduct research that directly addresses their needs.

## How SUPLN Benefits State Policymakers

A 2017 SUPLN survey found partnerships are largely involved in Medicaid policy analysis, with all partnerships engaging in Medicaid policy and program evaluation, and most focusing efforts on understanding the scope of issues facing Medicaid programs. (See Figure).

**Figure:**
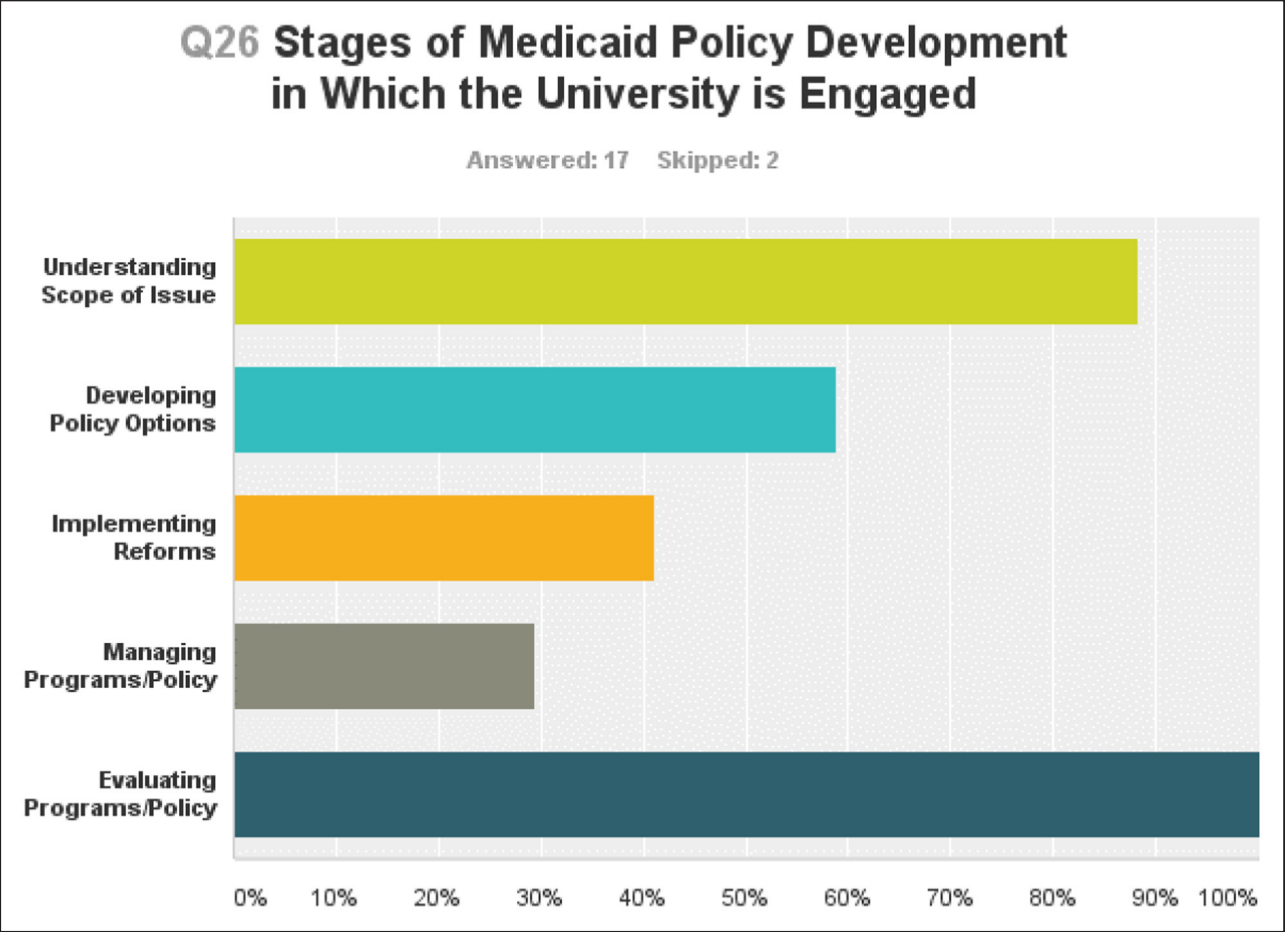


Partnerships conduct CMS quality improvement assessments, Medicaid benefit cost analyses, and analyses of specific beneficiary populations, such as pregnant women and those covered under Medicaid expansion. The following examples highlight the role SUPLN partnerships serve in generating research that informs Medicaid policy and practice demonstrating their collective capacity to provide Medicaid enrollee analysis within and across state Medicaid programs.

### Maryland

Since 1994, The Hilltop Institute at the University of Maryland, Baltimore County has maintained a collaborative and highly productive partnership with the Maryland Department of Health. In this capacity, Hilltop maintains an extensive data repository to support program development, research, policy analysis, and rate setting. For HealthChoice, Maryland’s Medicaid managed care program, Hilltop develops capitated payment rates for participating health plans, conducts the annual program evaluation for the Centers for Medicare & Medicaid Services (CMS), and performs policy research and analytics to support further program development. Hilltop developed and supports a decision support system for Maryland Medicaid with real-time data on eligibility, utilization, and expenditures. Hilltop managed development of *LTSSMaryland*—an integrated web-based information system that houses functional assessments, plans of care, and program enrollment information for Medicaid beneficiaries receiving long-term care. Hilltop links *LTSSMaryland* data to nursing home MDS data and Medicaid and Medicare administrative data to conduct analyses of the experience of individuals in the long-term care system.

### Pennsylvania

The Medicaid Research Center in the Health Policy Institute at the University of Pittsburgh, conducted research on the quality of treatment for opioid use disorder in Pennsylvania’s Medicaid program for the Pennsylvania Department of Human Services [6,7]. This research informed the monitoring and evaluation of health homes known as the Centers of Excellence, which help ensure that people with opioid use disorders stay in treatment to receive follow-up care and are supported within their communities. The centers coordinate care across providers, and treatment is team-based and “whole person” focused, with the explicit goal of integrating behavioral health and primary care [8].

### New Jersey

Rutgers Center for State Health Policy, working with the New Jersey (NJ) Medicaid agency and Housing and Mortgage Finance Agency, linked six years (2011–2016) of statewide Homeless Management Information System (HMIS) data with comprehensive Medicaid eligibility, encounter and claims data to compare patterns of health services utilization among Medicaid-enrolled persons who have experienced homelessness. Under Medicaid expansion, New Jersey Medicaid covers many more persons who have used homeless services. Through joint funding from NJ Medicaid and The Nicholson Foundation, the Rutgers team is comparing potentially avoidable Medicaid spending among homeless persons to demographically and clinically similar beneficiaries who have not used homeless services, and evaluating the impact of permanent supportive housing placements on Medicaid utilization and spending during the study period.

### Virginia

The Commonwealth of Virginia’s Department of Medical Assistance (DMAS) - Virginia Commonwealth University (VCU) partnership includes evaluations of the 2018 Medicaid Expansion, delivery and payment reforms for substance use under the Addiction and Recovery Treatment Services 1115 waiver, and Commonwealth Coordinated Care Plus programs. The DMAS-VCU partnership has adopted an embedded data analyst model to enable efficient data sharing between the two organizations. In this model, VCU employees serve as DMAS contractors and undergo data security training as well as trainings to learn to use the DMAS data warehouse to query the Medicaid data. Having the embedded VCU staff at DMAS enables real-time access to Medicaid files through VPN connections and secure DMAS-provisioned computers. This model ensures VCU analysts have the most current data DMAS has available to support critical Medicaid policy and program evaluations.

## Multi-state Medicaid Analyses: Value and Barriers to Implementation

Although university partners have become a critical asset for state Medicaid agencies, single state analyses have limited generalizability, due to variation across states in Medicaid benefit packages, eligibility criteria, payment systems, and other policies. Single state analyses are also limited in their ability to rigorously evaluate the effects of policies because they lack information on the counterfactual. Studying Medicaid one state at a time ultimately falls short of delivering on the promise of using state Medicaid programs as laboratories for testing health system innovations. At the same time, there are barriers to conducting multi-state analyses to inform Medicaid policy. States face numerous legal and regulatory restrictions to sharing data.

## Launching a Distributed Research Network

To support multi-state analyses of Medicaid data, increase the analytic rigor of policy evaluations in Medicaid, and contribute to the development of a Medicaid innovation laboratory, we formed the Medicaid Outcomes Distributed Research Network (MODRN) in 2017. A distributed research network is composed of multiple organizations using a common data model to support centralized development of analytic programs with local execution of analyses [9,10,11,12,13,14]. Members of SUPLN formed MODRN to support multi-state analyses of Medicaid data, thereby improving the external validity of Medicaid analyses [15,16], while maximizing security of sensitive health information. MODRN allows for standardization of analyses and sharing of aggregate results, but does not require the transfer of individual-level data. This obviated the need for multiple DUAs between states, which would be an almost insurmountable barrier. MODRN began in 2017 with 9 states (KY, MD, MI, NC, OH, PA, VA, WV, WI); two additional states (DE and TN) joined in 2019; and the network will expand to include additional states in the future. Critical organizational components of MODRN include a steering committee composed of state Medicaid agency staff, a Memorandum of Understanding between all participating state-university partnerships, and a Data Coordinating Center and Methods Core that provides overall analytic direction.

## Functions of a Distributed Research Network

MODRN executes 4 key functions of a distributed research network:

1. **Use of a common data model.** An essential function of distributed research networks is the use of a common data model. MODRN’s Data Coordinating Center develops the common data model and guides university partners in extracting, transforming, and loading their respective states’ Medicaid data into an analytic data set following the rules of the common data model. Each university’s resulting analytic data set has the same content, structure, variable names and formats. MODRN’s common data model includes five files: enrollment, inpatient, outpatient, professional, and pharmacy. The data elements contained in this common data model will support a variety of Medicaid studies and can be modified over time to accommodate new data elements.
2. **Standardization of analytic code.** Once the common data model is developed by participating universities, MODRN’s Data Coordinating Center distributes standardized programming syntax for the universities to use along with shell tables for aggregating results. Standardization of the analytic code facilitates efficient analyses of multi-state data and minimizes the likelihood of specification bias [17].
3. **Local analyses of Medicaid data.** SUPLN university partners conduct state Medicaid data analyses locally using the standardized analytic code. Each university obtains institutional review board (IRB) approval for research with their state’s Medicaid data, facilitates review of results by state Medicaid agency, and maintains control over data use and quality review.
4. **Data partners provide results, not data, to data coordinating center.** Each university partner submits aggregate results (not row- or person-level data) to the data coordinating center for aggregation, which presents results organized in a common template.

## MODRN – Opioid Use Disorder

The first research project to use the analytic infrastructure of MODRN focused on the quality and outcomes of treatment of opioid use disorder (OUD) among Medicaid beneficiaries. Medicaid programs play a significant role in addressing the opioid crisis, covering nearly 40 percent of individuals with an opioid use disorder [18]. Medicaid programs are taking a lead role in reforming substance use disorder treatment systems at the state-level. However, in spite of Medicaid’s important role in addressing the opioid crisis, there is a dearth of evidence about how quality and outcomes of OUD treatment vary across state Medicaid programs.

The MODRN-OUD project aimed to provide a comprehensive assessment of OUD treatment quality and outcomes in Medicaid in order to inform policy decisions about coverage of OUD treatments in Medicaid. To achieve this objective, MODRN-OUD identified 20 access, quality and outcome measures and constructed these measures using the MODRN analytic approach outlined above. A number of sources were consulted to identify potential measures for inclusion: 1) a 2017 CMS guidance to State Medicaid Directors outlining indicators of access to high-quality OUD treatment [19], 2) a comprehensive review of over 20 medication-assisted treatment guidelines (and associated quality measures) performed for the US Department of Health and Human Services in 2015 [20], 3) American Society of Addiction Medicine (ASAM) guidlines [21], and 4) the peer-reviewed literature [22,23,24,25,26,27]. MODRN-OUD findings point to significant variation in the prevalence of OUD, and in access and quality of treatment for OUD across state Medicaid programs.

## Limitations of a Distributed Research Network

While the benefits and potential of MODRN are exciting, we recognize the limitations within this model as well. Statistical analyses of distributed data require different approaches than those applied to pooled, individual-level data and we plan to build on approaches developed in other health DRNs [28,29]. We also expect that there may be challenges in adjusting for different benefits offered across the state, different ways some claims (e.g. ambulance transport) may be coded, or different ways that managed care organizations record encounters especially under capitated payment models.

## Suggestions for the Future

SUPLN offers myriad opportunities for rigorous individual and cross-state Medicaid policy analyses. From the use of federal matching to embedding researchers to establishing a sustainable distributed research network for timely cross-state collaborations, partnerships are striving to improve the quality of care for Medicaid beneficiaries.

### Future Applications of MODRN

The common data model, infrastructure, and analytic tools developed for the MODRN – Opioid Use Disorder project can be adapted for use in future Medicaid research by SUPLN partnerships. The data elements contained in the common data model can identify other clinical populations relevant to Medicaid, and construct measures of health care utilization and outcomes for evaluating health system interventions. Future projects of policy relevance to Medicaid include those focused on behavioral and physical health integration, telehealth, use of newer more costly biopharmaceuticals, evaluating state Medicaid waiver demonstrations including those with work requirements, and many others. Many SUPLN partners are currently engaged in single state analyses that link Medicaid data to vital statistics, housing data, income maintenance programs (i.e., Temporary Assistance to Needy Families), food and nutrition services (i.e., Women, Infant, and Children program; Supplemental Nutrition Assistance Program), child welfare system records (i.e., child protective services and foster care), and criminal justice records. The next frontier for MODRN will be to capitalize on these linkages to conduct multi-state analyses on the social determinants of health and evaluate opportunities to better integrate health and human services delivery.
